# Plasma HIV-1 RNA viral load rebound among people who inject drugs receiving antiretroviral therapy (ART) in a Canadian setting: an ethno-epidemiological study

**DOI:** 10.1186/s12981-016-0108-9

**Published:** 2016-07-25

**Authors:** Will Small, M. J. Milloy, Ryan McNeil, Lisa Maher, Thomas Kerr

**Affiliations:** 1 British Columbia Centre for Excellence in HIV/AIDS, St. Paul’s Hospital, 608-1081 Burrard Street, Vancouver, BC V6Z 1Y6 Canada; 2 Faculty of Health Sciences, Simon Fraser University, Burnaby, Canada; 3 Division of AIDS, Department of Medicine, University of British Columbia, Vancouver, Canada; 4 Kirby Institute, University of New South Wales, Sydney, Australia

**Keywords:** HIV, Injection drug use, Antiretroviral treatment, Treatment as prevention, Qualitative methods, Ethno-epidemiology, Risk environment

## Abstract

**Background:**

People who inject drugs (PWID) living with HIV often experience sub-optimal antiretroviral therapy (ART) treatment outcomes, including HIV plasma viral load (PVL) rebound. While previous studies have identified risk factors for PVL rebound among PWID, no study has examined the perspectives of PWID who have experienced PVL rebound episodes. We conducted an ethno-epidemiological study to investigate the circumstances surrounding the emergence of rebound episodes among PWID in Vancouver, BC, Canada.

**Methods:**

Comprehensive clinical records linked to a community-based prospective observational cohort of HIV-positive drug users were used to identify PWID who had recently experienced viral rebound. In-depth qualitative interviews with 16 male and 11 female participants explored participant perspectives regarding the emergence of viral rebound. A timeline depicting each participant’s HIV viral load and adherence to ART was used to elicit discussion of circumstances surrounding viral rebound.

**Findings:**

Viral rebound episodes were shaped by interplay between various individual, social, and environmental factors that disrupted routines facilitating adherence. Structural-environmental influences resulting in non-adherence included housing transitions, changes in drug use patterns and intense drug scene involvement, and inadequate care for co-morbid health conditions. Social-environmental influences on ART adherence included poor interactions between care providers and patients producing non-adherence, and understandings of HIV treatment that fostered intentional treatment discontinuation.

**Conclusions:**

This study describes key pathways which led to rebound episodes among PWID receiving ART and illustrates how environmental forces may increase vulnerability for non-adherence leading to treatment failure. Our findings have potential to help inform interventions and supports that address social-structural forces that foster non-adherence among PWID.

## Background

Antiretroviral therapy (ART) inhibits HIV viral replication and suppresses HIV plasma viral load (PVL) to undetectable levels, resulting in reductions in HIV-related morbidity and mortality, as well as reduced potential for onward HIV transmission [1]. Interventions promoting the preventive benefit of ART to reduce the onward transmission of HIV, known as Treatment as Prevention (TasP), have become a cornerstone of global HIV prevention efforts [2]. Due to the high rates of HIV infection among people who inject drugs (PWID), this population is a focus for TasP programs, but PWID often experience sub-optimal ART outcomes [3, 4], including high rates of treatment discontinuation and failure [5, 6]. Poor treatment outcomes among PWID have potential to undermine the effectiveness of TasP in preventing injection-related HIV infections [7], and comprehensive efforts to promote viral suppression in this population are required [4, 8].

While contemporary perspectives on HIV treatment among PWID increasingly emphasize the role of social-structural factors in shaping treatment outcomes [4], relatively few studies have investigated their impact on disease progression [9]. The HIV Risk Environment framework has been utilized to examine how HIV risk behaviours among PWID are shaped by interactions between individuals and social, structural, political, economic influences [10, 11], but has not yet been fully applied to examine the role various environmental forces play in shaping ART outcomes among PWID [9]. The Risk Environment framework has potential to help identify how broad social-level and structural-level influences constrain engagement with public health programs and impede individual ability to adhere to ART regimens [9–11]. While some evidence suggests that socio-economic marginalization, HIV-related stigma and discrimination, the criminalization of drug use, incarceration, and inadequate access to substance use treatment [9, 11], pose barriers to adherence, less is known about how these forces function to produce sub-optimal ART outcomes. Similarly, although drug use has often been identified as a risk factor for suboptimal adherence among injection drug-using populations [12, 13], recent research has suggested that PWID can achieve optimal adherence even in the context of ongoing drug use [14, 15]. This suggests that social-structural forces may play an important role in mediating the relationship between drug use and HIV treatment outcomes [9, 14, 15].

Plasma HIV-1 RNA viral load rebound (hereafter referred to as ‘rebound’), defined as the re-emergence of detectable plasma HIV viral load among individuals who had previously achieved viral suppression through ART, is a form of HIV treatment failure that represents a key challenge to the medical management of HIV [16, 17]. Approximately 10 % of all patients taking ART will experience a rebound episode [18], although it is not clear precisely how quickly viral load rebound emerge may due to missed doses of medication or treatment discontinuation given complex interactions between patient and virus characteristics, treatment histories, and regimen-specific effects. Shorter duration of viral suppression, particular ART medications, and non-adherence to treatment regimens are associated with increased risk of rebound, and viral rebound is a risk factor for the emergence of viral resistance, requiring less tolerable second-line and salvage-level therapies, and the onward transmission of transmission-resistant viruses [16, 18–20]. However, rebound appears to be far more common among PWID than other populations. A study from Vancouver, Canada, indicates that 45 % of PWID receiving ART displayed at least one rebound episode since initiating treatment [17]. In this study, rebound was associated with recent incarceration and involvement in sex work, while methadone maintenance therapy (MMT) was negatively associated with rebound episodes [17]. Importantly, when analyses controlled for adherence to ART, no measure of drug use was found to predict the emergence of rebound [17].

Due to the importance of long-term retention on ART and prolonged viral suppression for the effectiveness of TasP, there is an urgent need for greater understanding of the emergence of virologic failure among PWID [8, 17]. While risk factors associated with rebound among PWID have been identified, research to date has not examined the perspectives of PWID displaying rebound or documented the circumstances surrounding rebound events. Detailed knowledge of the lived experience of drug users can generate understanding of the production of specific health outcomes, and to add a deeper social dimension to conventional quantitative measures [21, 22]. Integrating targeted qualitative research with epidemiological inquiry, known as ‘ethno-epidemiology’ [23–25], has potential to improve knowledge regarding ART treatment outcomes by examining the experiences and perspectives of individuals who display particular outcomes. We conducted an ethno-epidemiological study to investigate the circumstances surrounding the emergence of rebound episodes among PWID in Vancouver, Canada, with a focus on the role of social-structural forces in the production of virologic failure. We examined the experiences and perspectives of PWID who had a recent episode of rebound through targeted qualitative interviews.

## Methods

This ethno-epidemiological study was nested within the AIDS Care Cohort to Evaluate Exposure to Survival Services (ACCESS), an ongoing prospective cohort of HIV-positive adults (≥18 years of age) with histories of illicit drug use (other than marijuana), which has been described previously [17]. Participants in this community-recruited cohort study provide a blood sample for virologic analysis and complete an interviewer-administered behavioural questionnaire at baseline and every 6 months thereafter. ACCESS data are linked with data collected by the Drug Treatment Programme of the British Columbia Centre for Excellence in HIV/AIDS, a province-wide centralized ART dispensary and clinical monitoring laboratory, providing a complete retrospective and prospective profile of CD4+ cell counts, PVL, and exposure to specific antiretroviral medications for all participants [17]. Universal healthcare is provided in this setting, and all HIV care (including ART) is provided free of charge, permitting examination of treatment outcomes among PWID in a context without financial barriers to access or medications.

**Fig. 1:**
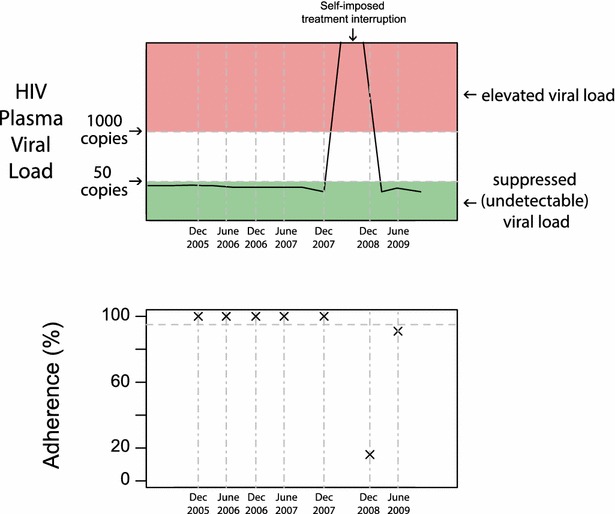
Sample clinical timeline graphic: detailing HIV plasma viral load and adherence over time for participant #12

In April 2011, we conducted a database query to identify ACCESS participants with recent rebound experiences. Inclusion criteria were all participants who: (i) were exposed to ART since enrolling in ACCESS; (ii) had at least two consecutive measurements indicating PVL suppression (<50 copies/mL) since 2007; and, had at least one measurement indicating elevated PVL (>1000 copies/mL) subsequent to PVL suppression. 43 individuals met these criteria, three of whom were recently deceased. For each participant, we generated a *clinical timeline graphic* (see Fig. 1) depicting PVL and adherence to ART over time. To estimate adherence, we used a validated measure based on pharmacy refill data [17], defined as the number of days ART was dispensed divided by the number of days since an individual was first dispensed therapy, capped at 180 days.

Potential participants were invited to participate in a qualitative interview when they visited or contacted the ACCESS cohort study office. Staff explained the study to potential participants and scheduled interviews. No one refused the offer to participate. We provided further information prior to the interview, and obtained written informed consent. Interviews were conducted with twenty-seven participants (see demographics in Table 1) between May 2011 and May 2012. Twenty-four interviews were conducted by the lead author, while three were conducted by a PhD student trained in qualitative interviewing.

**Table 1:** Characteristics of study participants

Characteristics	Interview participants *n* (%); n = 27
Age	
Median (min–max)	46.7 (32–54)
Gender	
Women	11 (40.74)
Men	16 (59.25)
Aboriginal ancestry	
Yes	11 (40.74)

A semi-structured interview guide was used to facilitate discussion regarding the circumstances surrounding rebound episodes, including HIV care, ART dispensing arrangements, drug use, living circumstances, and other contextual factors. A modified format of timeline interviewing, which is effective in linking individual narratives and experiences to wider social and environmental contexts [26], was used to retrospectively examine HIV treatment experiences in relation to each participant’s drug use career and personal biography, as well as to situate their experiences within their social-structural contexts. A simple timeline was hand-drawn by the interviewer during the interview, noting HIV care milestones and life events with the potential to impact upon ART adherence (e.g., housing transitions, changes in drug use patterns). This approach elicited extensive discussion of social-structural influences shaping adherence, as well as transitions between periods of adherence and non-adherence. The clinical timeline graphic was introduced at the conclusion of the interview to elicit further discussion of treatment events, summarize the interviewer’s interpretation of events, and allow participants to clarify inaccuracies. Some participants displayed more than one rebound episode over the study period, and all were examined during interviews and analysis. A total of 25 participants provided clear accounts of non-adherence related to their viral load rebound, as two participants were unable to provide a narrative regarding circumstances surrounding their rebound event due to problems related to recall.

Analysis focused on examining factors leading to prolonged interruption or discontinuation of ART. Interview transcripts and timelines were examined to understand each participant’s experience and rebound-related circumstances as an individual case, and a coding framework composed of a priori and emergent codes (e.g., housing transitions, drug use transitions, social isolation, understandings of ART) was then used to compare and contrast these cases. We also drew on the Risk Environment framework in our analysis [11], as well as the concept of structural vulnerability to illuminate how social and structural inequalities shaped engagement with care and adherence-related behaviour [27]. The analysis was conducted using word processing software to code, label, and retrieve data segments [28]. Ethics approval for the study was obtained from UBC/PHC Research Ethics Board. We have utilized the consolidated criteria for reporting qualitative research (COREQ) guidelines, to ensure full and accurate description of our study and analytical methods. [29] All names used in presenting the analysis are pseudonyms.

## Results

Our findings illustrate how rebound episodes were shaped by the complex interplay between individual-level factors and social, structural, and environmental forces operating within the HIV treatment risk environment. Participants had previously achieved viral suppression by maintaining optimal ART adherence, and their accounts emphasized how they had managed or adjusted their living situations, HIV care, ART dispensing arrangements, and drug use to ensure adherence through the development and maintenance of “stability” or regular “routines”. Rebound episodes were associated with disruption of these stable situations and routines, stemming from housing transitions, difficulty managing co-morbid health conditions, as well as changes in drug use and drug scene involvement. In addition, disruptions occurring due to poor relationships with care providers and misunderstandings regarding ART resulted in intentional treatment discontinuation in several cases.

### Impacts of housing transitions on ART dispensation and HIV care

For some participants, rebound episodes were temporally associated with changes in housing status and neighbourhood of residence, which negatively impacted HIV care. Housing transitions and neighbourhood relocations were particularly influential in disrupting established HIV care-related routines, and led to difficulty in re-establishing convenient ART dispensing arrangements. For example, Richard (participant #5, White Man, 52 years old) experienced a rebound episode after relocating to a neighbourhood approximately 5 km from where his HIV physician and pharmacy were located. He relied on a motorized wheelchair to travel to pick up his medications and his wheelchair began malfunctioning soon after moving. He was subsequently unable to pick up his HIV medications for more than 3 weeks, leading to his rebound episode.

HIV care and support services are concentrated in Vancouver’s urban core, and some participants experienced reduced access to HIV care, ART medications, and related supports after relocating outside the downtown core. Allen (participant #1, Afro-Canadian Man, 51 years old) experienced a disruption to adherence when he moved from the downtown core to a rural area to undergo addiction treatment at a residential treatment facility. He changed his pharmacy to pick up his monthly supply of ART medications closer to his new residence. Refilling his ART prescription required that he travel a considerable distance, and due to the lack of public transit and the regulations of the treatment facility he was attending, it was sometimes not possible to pick up his ART prescription until weeks after his supply of medication was exhausted. Difficulty picking up his ART medications resulted in non-adherence, which led to his rebound episode. Similar dynamics were evident in the experience of Bernadette (participant #10, Aboriginal Woman, 45 years old), who lived in a supported living residence for HIV positive individuals (including on-site ART dispensation), during the time she displayed viral suppression. Her rebound episode emerged soon after she relocated to a small town outside the city with her boyfriend. This change in residence reduced her access to HIV care and ART-related supports (e.g., daily dispensing), which previously facilitated optimal adherence, leading to non-adherence and, subsequently, her rebound episode. These accounts illustrate the interplay between location of residence and ART dispensing arrangements, and how disruptions associated with relocations can result in prolonged non-adherence and rebound.

### Relationships between extreme poverty, drug use, and non-adherence to ART

Regular ongoing illicit drug use was not reported to impede adherence, as many participants maintained viral suppression while continuing to use drugs. However, significant changes in drug use patterns were reported to hinder ability to take medications as prescribed in the context of extreme poverty and constrained opportunities for legal income generation. Participant accounts emphasized how increases in drug use, including episodes of binge use (often following the monthly distribution of social assistance benefits) and intense drug scene participation resulted in non-adherence leading to rebound episodes. Intense drug scene involvement was a key mechanism producing non-adherence, as participants reported that spending large amounts of time generating income (e.g., dealing drugs), and obtaining and using drugs disrupted adherence-related routines by keeping them away from home and their ART medication supply. The impact of these disruptions were exacerbated by various social, structural and environmental conditions related to the local drug scene, including difficulties generating income, the high costs of drugs within an unregulated market, and severe isolation due to a lack of social interactions outside the drug scene. Barriers to managing drug dependency were also evident in participant accounts and related to the impacts of changes in drug use patterns on adherence, including negative perceptions of regulations associated with MMT programs (e.g., observed daily dosing) and the lack of effective pharmacotherapies for stimulant use.

A recurring pattern of drug use binges was cited as playing a key role in producing multiple rebound episodes for Dan (participant #16, White Man, 48 years old), who described sustained binges that would result in multiple weeks of non-adherence. During periods of viral suppression, he reported using drugs in a more moderate manner. Episodes of binge drug use had a profound effect on his adherence due to the time and energy spent obtaining and using drugs. He attributed each of the rebound episodes in his clinical profile to prolonged drug binges:

*I went off it* [ART] *again cause I went on more on* [street] *drugs* […] *Each time where I’ve* [lost suppression]… *I’ve gone off on a binge.*

For other participants, intense drug scene involvement and the time spent generating income illegally and using drugs was perceived to negatively impact adherence due to the disruption of regular routines and time spent away from home and supplies of ART medications. Brendan (participant #2, White Man, 41 years old) reported spending large amounts of time in the drug scene to make money to purchase drugs, and then, after using stimulants for a prolonged period (often days), he would take a sleeping pill to “*crash*”. This resulted in numerous days each month when he would miss taking his ART due to the disruption of his normal routine (often coinciding with the payment of social assistance benefits). Similarly, Don (participant #21, White Man, 48 years old), described how his rebound episode emerged during a time when intense drug scene involvement resulted in an unstable lifestyle, which he contrasted with his stability and routines during periods of viral suppression. Instability stemming from drug scene involvement including illegal income generation (e.g., shoplifting), homelessness and repeated arrests and detentions, and difficulty managing drug dependency precipitated his rebound episode:

*I’ve always took my meds* [ART*], but for a few months there I was a little fucked up* […] *I wasn’t taking them* [ART] *responsibly.* [I was] *stopping and starting …*[due to] *the erratic lifestyle of like being homeless*.

While his adherence was briefly disrupted when incarcerated, he attributed his rebound episode primarily to the interaction of factors stemming from the instability of being homeless and entrenched in the drug scene.

### Inadequate care and support for comorbid conditions

Among participants with complex co-morbidities (e.g., mental health issues, chronic pain, opioid dependency), difficulties managing these conditions due to inadequate care and support led to disruptions in their regular routine and ART adherence, which contributed to rebound episodes. For Dave (participant #24, White Man, 49 years old), pain and reduced mobility shaped non-adherence by limiting his ability to pick up ART medications for self-administration. He experienced intense chronic pain from a spinal injury, and reported that his pain management was often inadequate. He had previously been prescribed morphine, which effectively managed his pain, but his physician had become reluctant to continue to provide this medication due to strict prescribing guidelines for opioid analgesics imposed by local medical regulatory body. Prior to his rebound episode, the pain from his spinal injury was unmanaged due to the discontinuation of his morphine prescription, which made it difficult for him to walk and, therefore, travel to his pharmacy to refill his ART prescription. Dave subsequently experienced a period of ART non-adherence lasting more than a month.

Inadequate care for ongoing and emergent mental health conditions was also critical in shaping non-adherence and rebound episodes. For example, Mary (participant #3, Aboriginal Woman, 37 years old) described how non-adherence to medications prescribed for her bipolar disorder often interfered with adherence to ART. Her rebound episode occurred when she was not taking both her “*psych meds*” and ART medications for a prolonged period because she felt depressed and suicidal. She reported that mental health services in the community did not provide her with adequate support, and she had to be hospitalised to receive care for her bipolar disorder. Meanwhile, because most participants were socially isolated, difficult life events (e.g., child apprehension, death of a family member) often led to serious depression accompanied by increased drug use, ART treatment interruptions, and rebound episodes. For example, Donna (participant #7, Aboriginal Woman, 35 years old) was forced to give up her baby to foster care soon after he was born, which led to deep depression, resulting in increased drug use and non-adherence to ART medications:

*I gave him* [her son] *up* [to foster care]. *I went onto the streets and started using heavy and got sick* [depressed]*…And stopped taking my meds* [ART].

Although she was engaged with services related to the foster care system, as well as HIV-related care, she did not receive adequate care for her depression, and self-medicated with illicit drugs. Similar dynamics were evident in the experiences of four other participants who reported that the death of family member resulted in depression accompanied by increased drug use. This relatively common sequence of events illustrates how interaction between life events, mental health issues, and changing drug use patterns precipitated rebound episodes among structurally vulnerable PWID who lacked mental health supports and strong support networks.

Difficulty managing opioid dependence, partially due to strict rules governing methadone therapy, resulted in MMT discontinuation and ART non-adherence for one participant. During the time that Laurie (participant #20, Aboriginal Woman, 38 years old) displayed viral suppression, she was receiving take away doses of methadone (“carries”) and daily-observed ART through her pharmacy. She reported being “cut-off” MMT carries when it was discovered that she was injecting her methadone and, since she was opposed to daily-observed methadone dispensation, she discontinued MMT. She subsequently began using large amounts of heroin and diverted methadone to manage her opiate dependence, and selling drugs to generate income. Managing opioid dependence by using illicit opioids rather than receiving MMT reduced her motivation to visit the pharmacy to pick up ART medications, resulting in non-adherence and rebound.

### Provider-patient interactions related to ART

Poor communication and interactions with HIV care providers negatively impacted individual adherence, and resulted in non-adherence and intentional treatment discontinuation among several participants. Calvin (participant #18, White Man, 55 years old) described how an intense disagreement with his physician led him to discontinue both ART treatment and MMT, which subsequently resulted in an episode of viral rebound. His physician expressed concerns regarding his adherence based upon reports from support staff at his residence stating that he had not been picking up his medications. He explained to his physician he had indeed been taking ART as prescribed, and that he had a stockpile of medications because he had not started his ART regimen until a month after receiving his first prescription. His physician demanded that he receive maximally assisted treatment/directly observed therapy (MAT/DOT) to address the perceived problem with adherence. He was vehemently opposed to observed therapy because he felt he was capable of being adherent and taking his medication independently:

Calvin: *I explained to her what I was doing* [adhering to regimen as prescribed by taking stockpiled medications]*, right… and she didn’t want to hear me. So, I ended up being off the medication* [ART] *and I got off methadone…*

Interviewer: *So she really wanted then for you to go every day and take the observed maximally assisted therapy?*

Calvin: *Exactly! I wasn’t gonna do that, no… Not at my age… I was over fifty years old. I know what I’m doing.*

The conflict with his physician caused him to leave care and discontinue ART treatment and MMT. He did not re-initiate ART until 24 months after this discontinuation event and remained off MMT for approximately 6 months, during which time he used heroin to manage his opioid dependence.

Similarly, Shelley (participant #26, Aboriginal Woman, 43 years old) described how she “*rebelled*” against her physician’s decision to put her onto MAT/DOT against her wishes by not adhering to her ART medication. She positioned observed therapy as paternalistic, emphasizing that she was “*not a kid*” and was indeed able to adhere to her regimen. She actively resisted her physician’s decision by not attending the MAT/DOT program to take her ART medications, which resulted in her rebound episode. These cases illustrate the impact of physicians’ decisions to have PWID patients receive directly observed therapy against their wishes, which unintentionally led to non-adherence and rebound because participants felt such arrangements were too paternalistic.

Negative interactions with care providers were also influential in the case of Marianne (participant #27, Aboriginal Woman, 41 years old), who was non-adherent for 3 months while incarcerated. Her ART medications were not available until 3 weeks after she arrived at a correctional facility. However, due to her frustration with the inability of institutional health care services to provide her medications, she refused to take her ART, despite awareness of the negative impact that this would have on her HIV treatment. She did not reinitiate ART until she was released to the community, and the 3 months of non-adherence while in correctional custody resulted in her rebound episode:

*I didn’t take it* [ART]. *I did* [served] *three months and then when I got out, I started taking it again.*

While the problems experienced by this participant partially stemmed from a lack of continuity in HIV care when in correctional custody, she reported that poor relationships institutional healthcare providers also led to her decision to intentionally discontinue her ART until she was released from jail.

### Local understandings of ART

Social influences and popular understandings of ART influenced participants’ beliefs regarding ART and, in turn, their adherence. Misunderstandings regarding adherence, management of ART resistance, and disease progression precipitated non-adherence to ART, as well as instances where patients intentionally discontinued their ART treatment. For example, Malcolm (participant #23, White Man, 49 years old) reported how ambivalence towards HIV treatment fostered his non-adherence, partially due to the understanding that intense drug use and ART were not compatible. During a period when he was using illicit drugs heavily, he reported low levels of motivation to adhere to ART medications as prescribed and experienced a rebound episode:

*You know when you’re not really into it [*ART treatment*], you don’t* [take your medications]*. I was still skipping doses and all that.I wasn’t really honest with myself* […] *I’d take them just when I thought I’d have to. Right? I had drawer full* [of ART doses he had not taken] *Fuck the medication*. […] *I was using fucking almost a half a eight ball* [1.75 grams] *of heroin a day*.

In a number of cases, patients intentionally discontinued their treatment despite awareness that they displayed undetectable viral load measures. Brett (participant #4, White Man, 53 years old) discontinued his ART intentionally due to concerns regarding missed doses, misunderstandings regarding ART resistance, and out-dated ideas regarding best practices in HIV treatment. He enacted a self-imposed treatment interruption because he believed stopping treatment would eliminate the potential for ART resistance and extend the effectiveness of his current ART regimen:

*I was on a structured treatment interruption*- *my own*. *I had missed enough adherence* [doses] *so… I went off for a year. And the doctor said why? I said… Instead of building a tolerance* [resistance] *it’s better if I go off it till I got no tolerance, and then we can put me back on it. So I did my own structured, my own treatment interruption.*

Similarly, Eric (participant #12, White Man, 53 years old) believed a treatment interruption might be a good way to prolong the efficacy of his current regimen, as he had begun ART treatment 19 years earlier and was concerned about long-term use of ART. His decision to discontinue ART, undertaken without consultation with his physician, was partially due to encountering information regarding strategic treatment interruptions in an old copy of a newsletter for HIV-positive individuals:

*I thought that a drug holiday would help. So I took myself off* [ART] *for one year and then…the viral load jumped up I took myself back on […] After more study they realized that it’s not good, but by that time I was already off of it so*…

This participant’s decision to discontinue treatment was partially shaped by his experiences with early treatment regimens, which influenced his understanding of contemporary therapeutic approaches. These intentional discontinuations occurred after structured treatment interruptions were no longer deemed clinically beneficial, and individual decisions to stop ART medications were not supported by physicians.

A lack of recognition that managing HIV with ART requires lifelong adherence to daily medication regimens reduced willingness to continue ART among some participants, leading to intentional treatment discontinuation. Darla (participant #9, White Woman, 56 years old) described her decision to discontinue ART, emphasising that taking numerous pills every day for years had become unappealing despite awareness that she had achieved viral suppression:

*I was undetectable for about two or three years. I just got tired of taking so many pills every day […]my viral load was right down and so I just said “I’m taking a break. I’m not gonna take it [ART] anymore. I’m just on too many pills.” […] It was something I just wanted to do and just kind of feel normal if I don’t have to take these pills every morning when I get up and again at night […] I just get sick of taking them.*

Similarly, Elaine (participant #15, White Woman, 50 years old) discontinued treatment when her viral load was undetectable, partially due to the belief that the positive outcomes she was experiencing (e.g., sense of well-being, weight gain) meant that she no longer needed to take ART to manage her HIV:

*I just got frustrated with taking them every day […] I started gaining weight and I started feeling good. I thought… “now I can do it on my own. Maybe I didn’t need it* [ART]*.” I just wanted to try to see if I can* [live without medication]*. But right away I started to crash, and I need the meds. I thought I was doing so good that I probably didn’t* [need medication], *I probably could do it on my own*- *I didn’t really need these pills anymore. And it’s not so, cause my body needs them… obviously.*

## Discussion

The findings of our study illustrate the role of interactions between individual-level factors and diverse social-structural forces operating within the HIV treatment risk environment in shaping the emergence of rebound episodes among PWID. Rebound episodes emerged when routines that facilitated adherence were disrupted by structural-environmental influences, including housing transitions, changes in drug use patterns and drug scene involvement, and inadequate care for comorbid health conditions. Social-environmental influences similarly disrupted participants’ adherence to ART as poor communication between care providers and patients precipitated non-adherence, and misunderstandings of ART functioned to reduce motivation to adhere to ART and produced intentional treatment discontinuation.

This study is the first to use qualitative and ethno-epidemiological methods to examine viral load rebound among PWID. Our findings generate new knowledge regarding how structural-environmental and social-environmental forces influence ART non-adherence among PWID, and lead to rebound episodes. While being homeless or marginally housed is a known risk factor for poor ART adherence and treatment outcomes among PWID [9, 30, 31], our findings indicate that housing transitions, including relocating to a different neighborhood, may influence treatment outcomes even among HIV-positive PWID who are continuously housed. These dynamics suggest that transitions in housing and relocation to different neighborhoods may be particularly influential among structurally vulnerable PWID, as living and care arrangements that function to enable optimal adherence may be easily disrupted. Changes in housing status and residence may also play a role in driving increases in drug use and involvement in illegal income generation among drug users [32], further underscoring the importance of maintaining housing stability among HIV positive PWID. Interventions designed to improve housing stability and quality may reduce the potential for non-adherence [33], but efforts to improve continuity of HIV care for individuals relocating their residence should focus upon ensuring specialist care and appropriate dispensing arrangements subsequent to housing transitions. Importantly, both supportive housing models and housing utilizing harm reduction supports appear to facilitate adherence among PWID given the documented beneficial effect on viral load [30, 34].

Recent research has challenged the perspective that illicit drug use directly contributes to poor ART outcomes [9], highlighting the importance of ART adherence in mediating the relationship between drug use and treatment outcomes [14, 15]. Our results underscore the importance of understanding how extra-individual forces (including physical and psychological comorbidities) interact with varying patterns of drug use to shape non-adherence [9], and elucidate how changes in drug use and drug scene involvement impede individual ability to adhere by disrupting adherence-related routines. Intense drug scene exposure has previously been associated with poor health outcomes and more severe substance use [35], and our findings suggest that time spent in the drug scene (generating income and using drugs) represents a mechanism through which drug use shapes non-adherence. Given that intensity of drug scene involvement and illegal income generation may mediate the relationship between drug use and ART adherence, the potential of alternative income generation and low-threshold employment interventions for PWID living with HIV should be explored [36, 37]. Improving economic opportunities may represent an effective strategy to reduce or limit drug use and drug scene involvement [32], and employment has been previously documented to positively impact ART adherence [38].

While MMT has been associated with optimal adherence [39–41], our findings illustrate how the benefit of MMT upon ART adherence can be eroded and negated among PWID. Specifically, we found that MMT discontinuation events also coincided with non-adherence and viral rebound. An established literature shows that restrictive programmatic features of MMT can result in suboptimal outcomes and poor treatment retention [42, 43], and additional supports for HIV-positive PWID should be considered, including low threshold MMT, outreach to individuals who discontinue MMT, and alternatives to MMT (including heroin prescription programs and buprenorphine). These supports might help facilitate optimal adherence over the long-term among PWID.

Inadequate support across the continuum of care for PWID living with HIV with comorbid conditions (e.g., mental health conditions) resulted in non-adherence and rebound. While it is recognized that medical and psychiatric comorbidities among HIV-positive PWID (and the concurrent nature of these conditions) contribute to poor ART outcomes [44], appropriate interventions are lacking in most settings. The role comorbid conditions played in disrupting adherence among study participants illustrates the importance of comprehensive care and support for HIV positive PWID in maintaining optimal adherence. There is a need for integrated health services, including multi-component and interdisciplinary approaches, delivering integrated case management to simultaneously improve the management of comorbid conditions and adherence to ART [44–47].

In the context of inadequate linkage with mental health services, existing and emergent mental health conditions affected adherence, illustrating the importance of enhanced care and support for mental health conditions among HIV-positive PWID. Interventions to manage mental health conditions among PWID include community-based integrated mental health services programs, social supports, peer-support programs, and housing-based interventions [48, 49]. Unfortunately, in many settings (including Vancouver), biomedical and legal approaches to the management of mental illness have become increasingly dominant, detracting attention from social and structural forces driving suboptimal treatment of mental health conditions, and eroding support for community-based programs [48]. Detainment of individuals diagnosed with mental illness produces negative effects on adherence and treatment outcomes [4]. The utilization of peer-based interventions [50, 51], including peer-counselling, peer navigators, and peer-support programs, could contribute to the effective management of mental illness and simultaneously support optimal adherence among PWID [52–54]. Local drug user groups may be a vehicle for mobilizing community resources to promote social support and engagement with care [55].

Our findings also indicate that poor communication and interactions between care providers and patients precipitated discontinuation of ART, particularly in relation to physician decisions regarding the necessity of MAT/DOT programs to ensure adherence among PWID. While MAT/DOT programs are associated with improved adherence and increased likelihood of viral suppression [56], treatment decisions should be jointly made by patients and physicians [57], and with the understanding that some participants perceive observed therapy to be paternalistic. While our findings are consistent with past work showing that incarceration may shape non-adherence through treatment interruptions stemming from delays in obtaining medications while in custody [6, 58], poor relationships with institutional health care providers may also lead PWID to discontinue ART while incarcerated. These dynamics have previously been documented in our setting [59], and PWID routinely re-initiate treatment upon return to the community, although some do experience challenges obtaining medications and connecting with physicians post-release. As noted previously, correctional services have a duty to provide essential health care in a timely manner, including ART medications, and such efforts are essential to ensuring continuity of HIV care for PWID who experience incarceration [59]. Improving relationships and communication between PWID and physicians may benefit adherence [60], and efforts to improve patient-provider interactions may involve better training for physicians who care for PWID, as well as education regarding addiction medicine and substitution therapies for opioid dependence [61, 62].

Understandings of HIV treatment played a role in shaping adherence to ART among study participants. Our findings illustrate how numerous years of ART may accumulate to make individuals “tired” of pills given a lack of appreciation that managing HIV with ART requires lifelong adherence. This illustrates how treatment fatigue plays a role in shaping intentional treatment discontinuation, as qualitative examination of these issues has been lacking to date [63]. While increasingly simple regimens involving fewer pills may reduce levels of fatigue, ensuring long-term retention of PWID will require further interventions to prevent intentional discontinuation and that care providers emphasize the lifelong nature of ART when communicating with patients [64]. In addition, individuals may want to have a sense of control over their HIV treatment and medications, which may entail stopping or modifying treatment regimens (particularly in the context of long-term ART), and these dynamics may further complicate communication between patients and care providers. Our findings document how particular beliefs regarding resistance and disease progression, as well as anachronistic ideas regarding best practices in HIV treatment, played a role in producing intentional treatment discontinuation. This is particularly significant given emerging clinical research suggesting that rates of antiretroviral resistance among PWID are less of a concern than previously thought [65], highlighting the importance of care providers delivering ongoing education and accurate information about HIV treatment [66]. While improving patient-provider communication is an important strategy for correcting misconceptions regarding ART, social models of education and information provision (similar to peer-led safer injection education) might also improve understandings of ART [55], correct outdated beliefs, and address common concerns regarding long term retention in HIV treatment [67].

The current study has several limitations. Our findings are specific to the experience of individuals participating in the study, and other PWID displaying viral rebound may have different experiences and perspectives. Some participants were not able to describe circumstances surrounding the emergence of rebound episodes, and our analysis has placed emphasis upon cases where participants were able to provide a complete account of the emergence of rebound episodes. While the design of our study allowed for direct examination of the experiences of PWID who recently displayed rebound, further research regarding circumstances surrounding rebound is needed.

## Conclusions

In conclusion, this study identified pathways leading to viral rebound episodes among PWID who had achieved viral suppression, and illustrates the impact of environmental forces in shaping non-adherence and treatment failure. Our findings highlight the need for interventions and supports that address the social-structural forces that create vulnerability to ART non-adherence among PWID.

## Abbreviations

ACCESS: AIDS care cohort to evaluate exposure to survival services
ART: antiretroviral therapy
COREQ: consolidated criteria for reporting qualitative research
MMT: methadone maintenance therapy
MAT/DOT: maximally assisted treatment/directly observed therapy
PVL: HIV plasma viral load
PWID: people who inject drugs
TasP: HIV treatment as prevention
